# Effects of light emitting diode irradiation on neural differentiation of human umbilical cord-derived mesenchymal cells

**DOI:** 10.1038/s41598-017-10655-w

**Published:** 2017-08-30

**Authors:** Samereh Dehghani-Soltani, Mohammad Shojaee, Mahshid Jalalkamali, Abdolreza Babaee, Seyed Noureddin Nematollahi-mahani

**Affiliations:** 1 Department of Anatomy, Afzalipour School of Medicine, Kerman University of Medical Sciences, Kerman, Iran; 2 Afzal Research Institute, Kerman, Iran; 3 Semiconductors Group, Photonics Research Center, Graduate University of Advanced Technology, Kerman, Iran; 4 Kerman Neuroscience Research Center, Institute of Neuropharmacology, Kerman University of Medical Sciences, Kerman, Iran

## Abstract

Recently, light emitting diodes (LEDs) have been introduced as a potential physical factor for proliferation and differentiation of various stem cells. Among the mesenchymal stem cells human umbilical cord matrix-derived mesenchymal (hUCM) cells are easily propagated in the laboratory and their low immunogenicity make them more appropriate for regenerative medicine procedures. We aimed at this study to evaluate the effect of red and green light emitted from LED on the neural lineage differentiation of hUCM cells in the presence or absence of retinoic acid (RA). Harvested hUCM cells exhibited mesenchymal and stemness properties. Irradiation of these cells by green and red LED with or without RA pre-treatment successfully differentiated them into neural lineage when the morphology of the induced cells, gene expression pattern (nestin, β-tubulin III and Olig2) and protein synthesis (anti-nestin, anti-β-tubulin III, anti-GFAP and anti-O4 antibodies) was evaluated. These data point for the first time to the fact that LED irradiation and optogenetic technology may be applied for neural differentiation and neuronal repair in regenerative medicine.

## Introduction

In the recent years, the physical factors such as light irradiation emitted from different sources including light emitting diodes (LEDs), have been introduced as a potential physical factor for proliferation and differentiation of various cell types^1–3^. Peng *et al*. reported that proliferation of bone marrow mesenchymal cells can be enhanced by LED irradiation^4^. Also, Li Wen *et al*. have shown that red light promotes osteogenic differentiation of rat bone marrow mesenchymal cells^5^. The mechanism involved in cell proliferation and maturation following light irradiation is not fully revealed, but it has been shown to be associated with an increase in oxidative function of mitochondria^6, 7^. In addition to mitochondria enhancement, red and green lights could affect other biological phenomena and promote cell differentiation^8–10^.

Mesenchymal stem cells (MSCs) have been introduced as an effective and appropriate source of stem cells for the treatment of various diseases including neural disease^11–13^. Human umbilical cord matrix-derived mesenchymal (hUCM) cells show unique features (e.g. ease of isolation, self-renewing properties and shorter population doubling time compared with bone marrow stem cells) that make them more appropriate for regenerative medicine procedures^14–16^. Additionally hUCM cells exhibit reduced immunogenicity especially at lower passages^17^ and can be induced to generate different type of cells, such as adipocytes, osteocytes^18^, myocytes^19^, hepatocytes^20^, and neurons, oligodendrocytes and other glial cells^21–23^. These cells have also been successfully used for the treatment of acute myocardial infarction without elevation of immune responses^24^. Although some studies have been conducted so far to introduce different biological exogenous factor for neural lineage differentiation^25–28^, there is still no extensively accepted method for neural differentiation of mesenchymal stem cells, including hUCM cells. Retinoic acid (RA) has long been introduced as a powerful exogenous neural inducing factor for neural differentiation with and without other inducing components^13, 29, 30^.

Enhancement of stem cells differentiation capacity is important for further advance of cell therapy and have a key role in regenerative medicine^31^. Thus, we aimed at the present study to investigate the effect of red and green light irradiations (provided by appropriate LEDs) on the neural lineage differentiation of hUCM cells in the presence or absence of RA as a known chemical inducing factor.

## Results

### Features of hUCM cells

**Figure 1:**
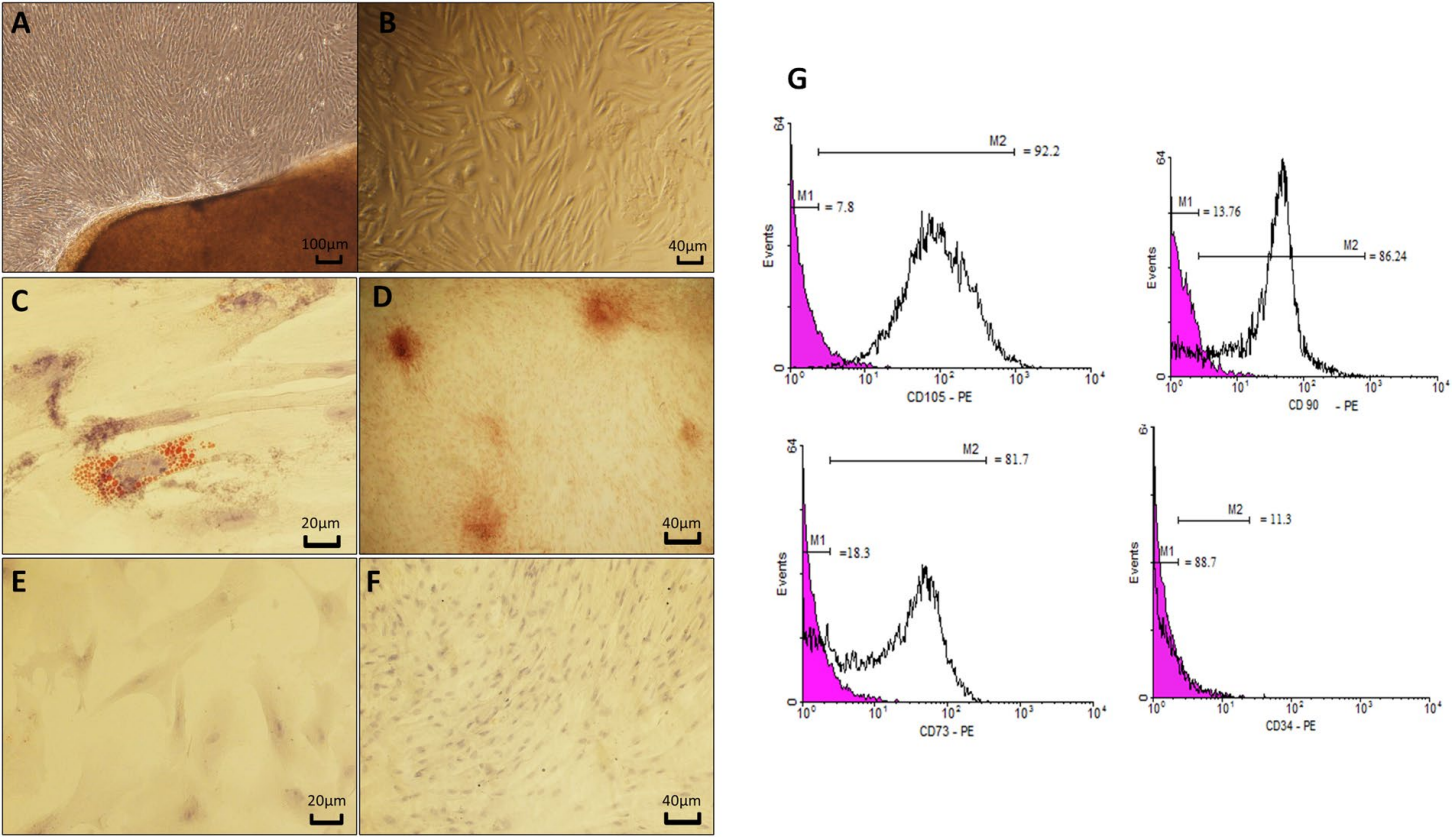
Projection of hUCM cells from boundary of the Wharton’s jelly fragment, 12 days after culture (**A**). Fibroblast-like and spherical morphology of hUCM cells (**B**). Adipogenic and osteogenic differentiation of hUCM cells. (**C**) Oil Red O staining displayed lipid containing vacuoles, after culture in adipogenic medium. (**D**) Formation of calcium deposits via osteogenic induction (Alizarin Red staining). None induced hUCM cells were used as control for adipogenic (**E**) and osteogenic (**F**) differentiation. Immunophenotype of hUCM cells was examined by flow cytometry. hUCM cells were negative for the hematopoietic marker (CD34), while strongly positive for mesenchymal stem cell specific markers including CD73, CD90, and CD105. The black histograms represent antibody labeled cells and colored histograms show isotype control cells.

After 8–10 days, fusiform and fibroblast-like cells appeared at the boundary of Wharton’s jelly fragments (Fig. 1A and B), after which the explant segments were removed (at day 12 of culture). Some of the propagated cells exhibited round shape while the majority was elongated with cytoplasmic processes. Mitotic index was abundant in the culture (data not shown).

### Adipogenic and osteogenic differentiation capacity of hUCM cells

A Collection of lipid vacuoles in the cytoplasm of induced cells was discovered with Oil Red O staining after 18 days of adipogenic treatment (Fig. 1C). In addition, calcium phosphate deposits in extracellular matrix of osteogenic induced cells were detected by means of Alizarin red staining (Fig. 1D). No Oil Red O or Alizarin red-positive cells were detected in the control groups (Fig. 1E and F).

### Flow cytometry and surface marker assessment

The isolated cells were assessed for hematopoietic as well as MSC markers. Results showed that hUCM cells did not express CD34 (as hematopoietic cell marker) while they strongly expressed MSC markers; CD105, CD90 and CD73 (Fig. 1G).

### Immunocytochemistry

**Figure 2:**
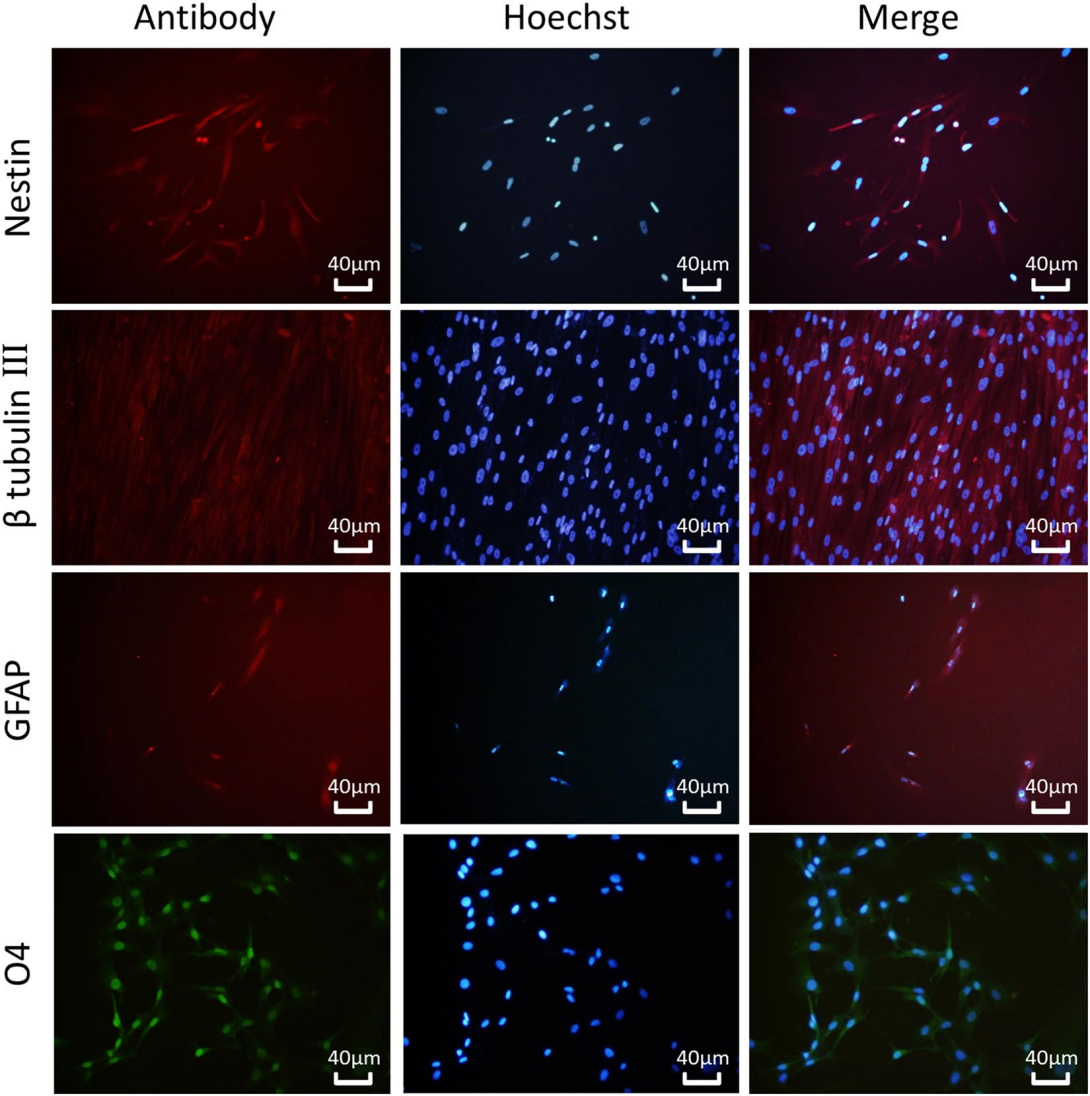
Immunocytochemical analysis of Nestin, β tubulin III, GFAP and O4 proteins after neural induction of hUCM cells. The cell nuclei were counterstained with Hoechst.

**Figure 3:**
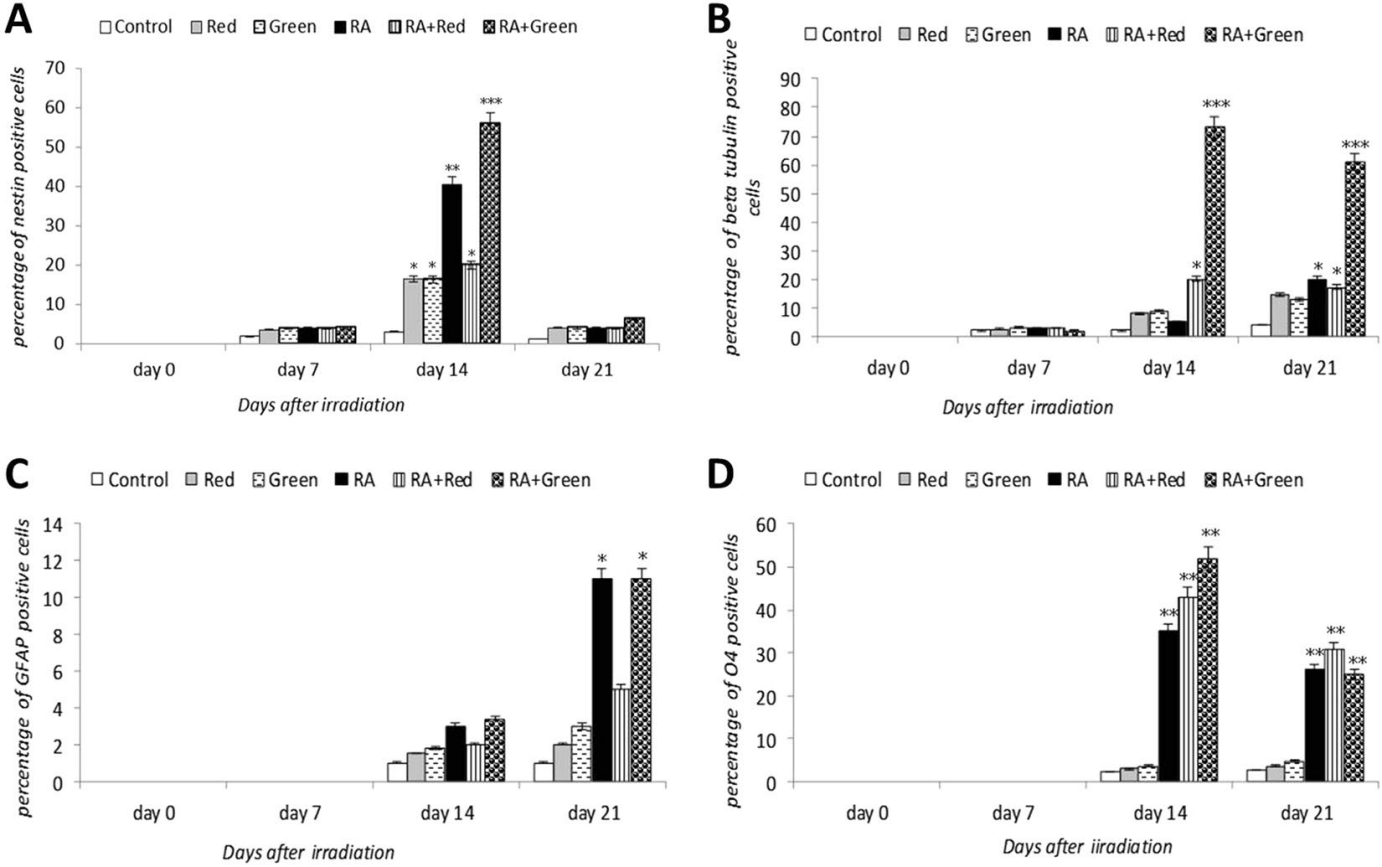
Immunofluorescence of neural lineage markers in the different groups. presence of Nestin (**A**), β tubulin III (**B**), GFAP (**C**) and O4 (**D**) proteins was evaluated on days 0, 7, 14 and 21 after irradiation. Data were pooled from three independent experiments and were expressed as the mean ± SD. (*p < 0.05, **p < 0.01, ***p < 0.001).

At days 0, 7, 14 and 21 after the onset of treatment, the presence of neural specific proteins including nestin, β-tubulin III, GFAP and O4 in all groups was evaluated by immunocytochemical analysis (Fig. 2). Nestin (an intermediate filament in neural precursor cells) production increased to a significant level in the induced hUCM cells on day 14 of induction but decreased toward the end of induction period (day 21, Fig. 3A). Highest expression of nestin was observed in Green + RA group followed by RA group. Interestingly, the cells in the Red and Green groups also exhibited more nestin positive cells compared with the control group. Expression of β-tubulin III, an intermediate filament which appears in the mature neuron like cells, was also significantly higher in Red + RA and Green + RA groups on day 14, with a sharp increase in Green + RA group. It also remained high in Green + RA, Red + RA and RA groups on day 21. The cells in the Green and Red groups gradually expressed more β-tubulin III during the culture period but did not reach a significant level when compared with the control group (Fig. 3B). GFAP, an intermediate filament specific in glial cells did not express until the day 14, after which it was expressed higher than control on day 14 in all the experimental groups and reached a significant level on day 21 in RA and Green + RA groups (Fig. 3C). O4, an intermediate filament in oligodendroglia cells did not express before day 14 in either of the experimental groups. However it expressed thereafter and reached a significant level on day 14 in Red + RA, RA and Green + RA groups compared with the control group but decreased to a lower level on day 21 (Fig. 3D).

### Light microscopic study of differentiated hUCM cells

**Figure 4:**
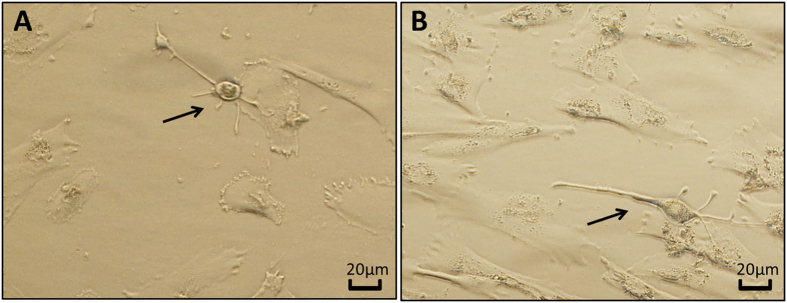
hUCM cell-derived neuron- like cells (arrow) after 10 days of incubation with RA (**A**) and after 18 days in the green light irradiated group.

All groups were observed daily by a light microscope after induction of neural differentiation in hUCM cells. The microscopic examination demonstrated that after 6–9 days in RA, RA + Green and RA + Red groups and after 14–16 days in Green and Red groups, some of the cells appeared irregular in shape and subsequently acquired neuronal shape (Fig. 4). However, cells in the control group exhibited a fibroblast-like shape through the experiments.

### Evaluation of neural specific gene expression

**Figure 5:**
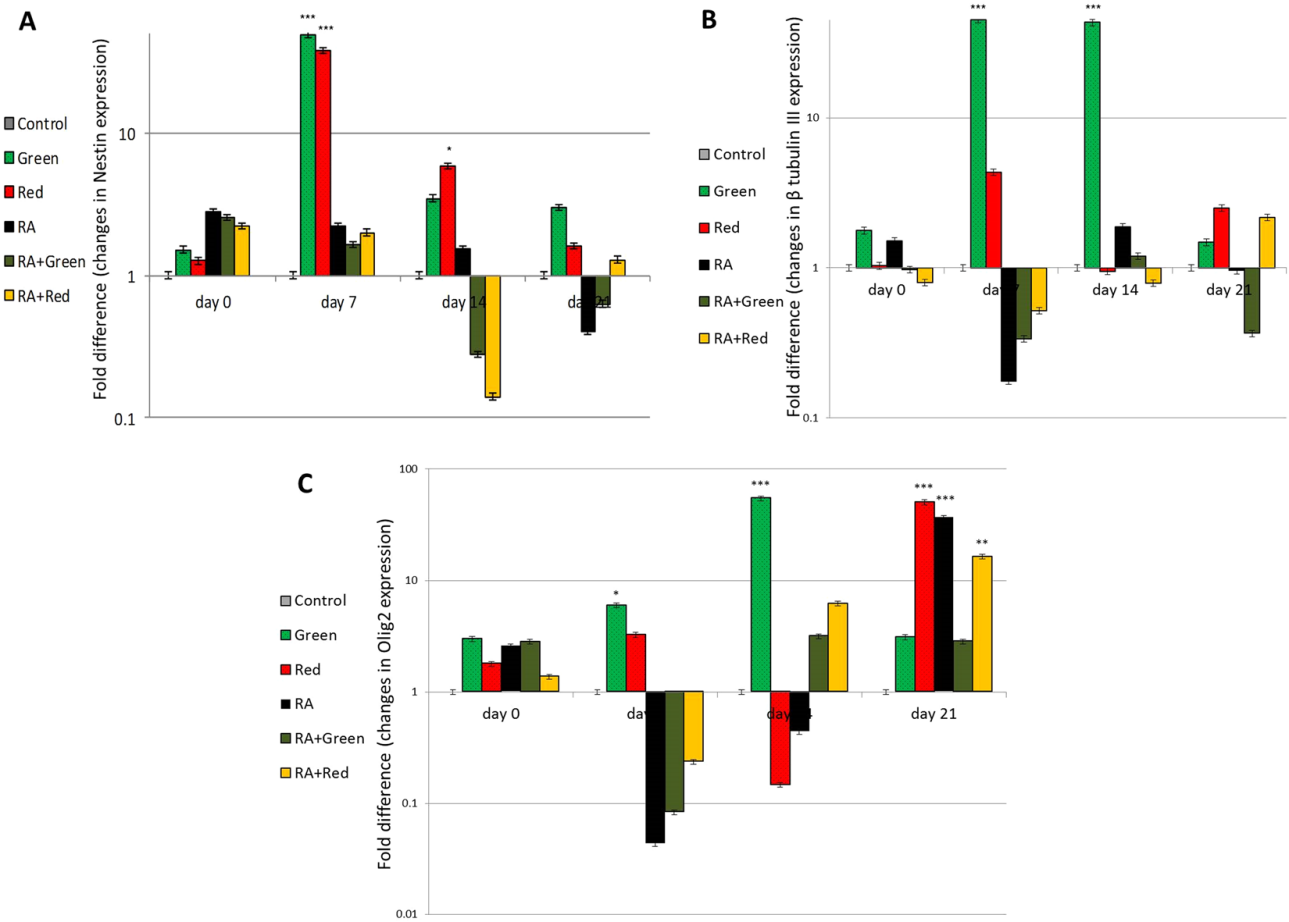
q RT-PCR analysis. Histograms displayed hUCM cells expression of Nestin (**A**), β tubulin III (**B**) and Olig2 (**C**) markers in Green, Red, RA, RA + Green and RA + Red groups, relative to the control group (on days 0, 7, 14 and 21 after irradiation). Results are the mean ± SD of three independent experiments (*p < 0.05, **p < 0.01, ***p < 0.001). Notice that the data were shown in logarithmic scale and the values below the base line are not actually negative values.

We analyzed the gene expression of known neural markers including nestin, β-tubulin III and Olig2 by qRT-PCR in the induced hUCM cells and controls at days 0, 7, 14 and 21 after LED irradiation. The results, presented in Fig. 5, show that after green and red LED irradiation, nestin expression increased significantly on day 7 and continued to be high at day 14 but it decreased to a nonsignificant level at day 21. Green LED irradiation also resulted in a profound effect on β-tubulin III expression on days 7 and 14 compared with the control group. However, prolongation of culture period to 21 days reversed the effects. Red LED irradiation also caused induced hUCMs to express more β-tubulin III gene at days 7 and 14 but it did not reach a significant level when compared with the control group. Other groups (RA, Green + RA and Red + RA) did not express β-tubulin III significantly at either time points compared with the control group (Fig. 5). Green LED irradiation caused hUCM cells to express significantly higher level of Olig2; a gene involved in motor neuron and oligodendrocyte differentiation, at days 7 and 14 and a nonsignificant level at day 21. A significant increase in Olig2 gene expression was detected in RA, Red + RA and Red groups at day 21 compared with the control group (Fig. 5).

## Discussion

In the current study, the effect of green and red lights on neural differentiation of hUCM cells was investigated. Some studies have reported that LED irradiation could improve cell proliferation^1, 5, 32^. Kim *et al*. have shown that LED irradiation enhances osteogenic differentiation of mesenchymal stem cells^33^. We could demonstrate that LED irradiation with or without RA pre-treatment enhances neural differentiation of hUCM cells, especially when neural-specific gene expression was considered. In the recent years, studies have introduced different factors and conditions effective in neural differentiation of mesenchymal stem cells, including various concentrations of RA, epidermal growth factor (EGF), fibroblast growth factor (FGF) and utilization of 3D scaffolds^13, 34, 35^. Our finding highlights a new interesting strategy for the induction of MSCs into neural lineage that requires more attention to understand the precise mechanisms involved in neural lineage differentiation.

A previous study reported that green light (530 nm) irradiation increases the neural sprouting in neuroblastoma cells. The proposed method could assist in phototherapy usage for improving sprouting and promoting neural network formation^36^. Our findings demonstrated that red and more specifically green light (530 nm) irradiation, in the absence of retinoic acid, stimulates neural lineage differentiation of hUCM cells.

The way that light emitted by LEDs interacts with the cells and tissues depends on the physical characteristics of light, mainly the power density, exposure time and wavelength. In addition to the light characteristics, the type of cell may be impressive^3, 9^. The mechanisms which are involved in cellular differentiation and proliferation following LED irradiation is very complicated and yet to be understand well. Some studies have proposed that it may be associated with an increase in cellular ATP and activation of ERK signaling pathway^6, 8^. When green light (530 nm) was applied on mesenchymal stem cells isolated from orbital fat tissue the motility and migration capacity was altered^8^. Also, low level laser irradiation (810 nm, 3 and 6 J/cm^2^) improved differentiation of bone marrow mesenchymal stem cells into osteoblast and neurons (in the presence of bFGF)^37^. Compared to lasers, LED devices are light-weight and cost-effective. Some investigations have claimed that LEDs are more effective than lasers for photodynamic therapy and photo-stimulation^38, 39^. However, based on other reports this issue is controversial and depends on the irradiation parameters, especially dose and wavelength^40, 41^.

In agreement with Eftekhar-Vaghefi^23^ and Bagher^34^ studies we have demonstrated that nestin is a leading protein in the sequence of neural differentiation (day 7), when compared to the other neural markers. We could show that induction of hUCM cells by light irradiation improved glial cell differentiation via expression of glial cell markers; Olig2, GFAP and O4. Deisseroth *et al*. reported that membrane depolarization and calcium waves have a vital role in the neural differentiation of adult neural stem/progenitor cells^42^. Also, Stroh *et al*. introduced an optogenetic stimulation method for modulation of stem cells differentiation via control of Ca^2+^ ion flux^43^. Therefore, in our study it is likely that, LED irradiation affected the membrane polarization and/or Ca^2+^ ion flux. Previous studies reported that RA by means of several cell signaling pathways including JNK/CREB, AKT/CREB and phosphorylation of ERK1/2 improved neural differentiation of stem cells^44, 45^. Also, Park *et al*. reported that electromagnetic fields can improve neural differentiation through EGFR activation which is mediated by ROS production^46^. However, it is unclear whether LED induced neural-lineage differentiation might follow the same pathways or not. We suggest that investigation of ATP production, Ca ion changes in the cytoplasm and ROS production at different time points after irradiation, as well as assessment of phosphorylation of proteins that are involved in the cell signaling of neural differentiation, might clear the molecular mechanisms underlying stem cells neurogenic differentiation. With this approach in mind, optogenetic knowledge may highlight appropriate methods in stem cell biology and cell therapy. Whether, other light wavelengths, single or multiple exposure, and duration of exposure would change the differentiation capacity of mesenchymal cells needs to be examined in the further studies.

## Conclusions

Together, the results of our study indicate that the given dose of red and especially green LED irradiation on hUCM cells could successfully enhance neurogenic gene expression. However, when neural lineage differentiation proteins production is considered, red and especially green led irradiation could enhance RA effects on nestin, β-tubulin III and O4 after 14 days and GFAP after 21 days. Because light stimulation is dose dependent and the penetration of light beam in different tissues is variable, it is not possible to extend *in vitro* results directly to *in vivo* applications. However, as LED irradiation is economic, safe and easy to use, our results suggest that further studies in the animal models can lead to possible application of LED irradiation as a useful tool for promotion of neuronal repair and nerve regeneration.

## Materials and Methods

All the materials used in this study were purchased from Sigma Company (Sigma-Aldrich, MO, USA) unless those stated otherwise. Institutional ethical review board of Kerman University of Medical Sciences, Kerman, Iran, approved the study.

### Isolation and culture of hUCM cells

We used a previously reported protocol^47^ for the isolation and culture of hUCM cells with minor modifications. Briefly, Wharton’s jelly, obtained from fresh human umbilical cords, was cut into 2 to 3 mm pieces and cultured in DMEM/F12, supplemented with 10% FBS (PA Biologicals, Sydney, Australia), 100 IU/ml streptomycin, penicillin and 2 µg/ml amphotericin B. The culture Petri dishes (Falcon BD, Franklin Lakes, NJ, USA) were incubated at 37 °C in the humidified air with 5% CO_2_. The medium was refreshed every 72 h. The culture continued until the cells reached 80% confluence. hUCM cells at Passage 2 to 4 were used for the experiments. Also, some of the viable cells were cryopreserved with conventional freezing protocols.

### Cell marker analysis by flow cytometry

To assess surface antigen expression, 2 × 10^5^ viable cells at passages three were harvested by trypsinization. The cells were washed with PBS and fixed by 10% formaldehyde for 15 min. After centrifugation and washing, the cells were incubated with 10% goat serum in phosphate-buffered saline (PBS) for 20 min to block nonspecific binding sites^48, 49^. The cells were incubated for 1 h at 4 °C with following phyco-erythrin (PE)-conjugated antibodies: CD34, CD105, CD73 (Chemicon, Temecula, CA) and CD90 (Dako, Glostrup, Denmark). In the control group, the cells were stained with PE-conjugated mouse IgG isotype antibody. At least 12,000 measures were recorded for each sample with FACS Canto flow cytometer machine (BD Biosciences, San Jose, CA) and data were analyzed by WinMDI software (West Lafayette, IN. USA).

### Adipogenic and osteogenic differentiation

To assess differentiation capacity of the isolated cells, third passage hUCM cells at a density of 2.5 × 10^4^ cells/cm^2^ were seeded onto glass slides with DMEM/F12 supplemented either with adipogenic (50 μg/ml indomethacin and 100 nM dexamethasone) or osteogenic (10 nM dexamethasone, 10 mM β-glycerophosphate and 50 μg/ml ascorbic acid,) differentiation medium for 18 days. The culture media were refreshed every 72 h. Adipogenic and osteogenic differentiations were detected with Oil red O and Alizarin red staining, respectively^1, 49^.

### Light irradiation

**Figure 6:**
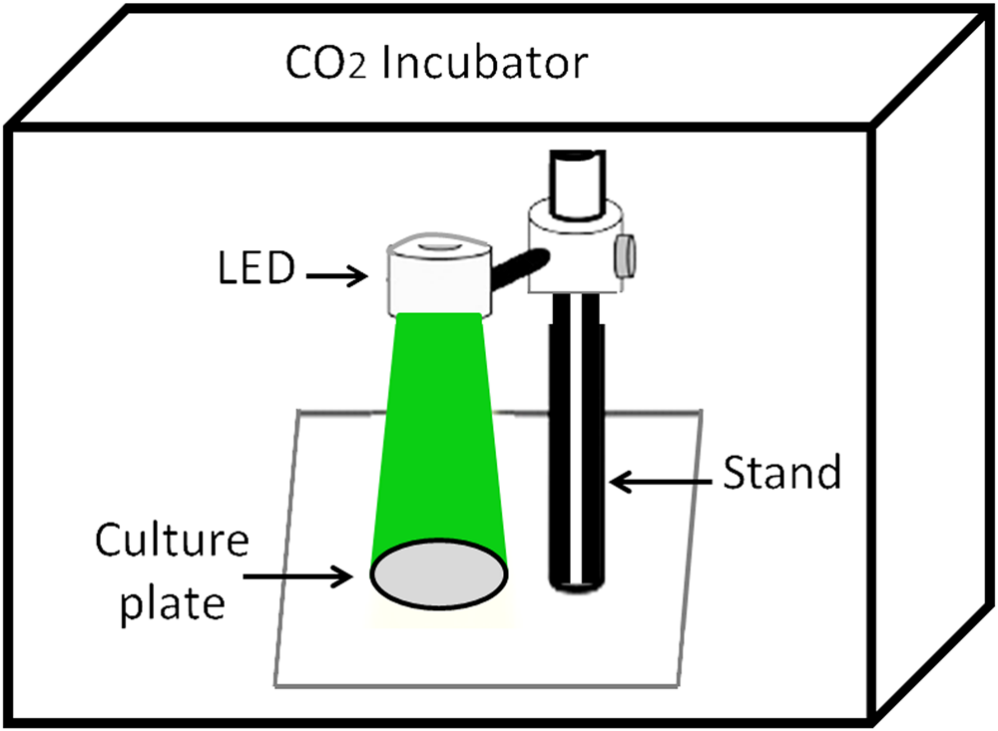
A schematic picture of LED device used in this investigation. All the exposures were carried out inside a CO_2_ incubator that was used for irradiation only. Each culture plate was exposed to different irradiations, separately. While other culture plates were incubated in another CO_2_ incubator.

Handmade LED devices were used as light sources (Fig. 6). Each of these devices consisted of red (630 nm with 10 nm bandwidth) or green (530 nm with 20 nm bandwidth from SE Electronics, China) lights. The LED array was planned to fit into 3 cm culture plates. These plates were divided into the control and treated groups (Green, Red, RA, RA + Green, RA + Red). The power density and distribution of LED array radiation was measured by appropriate meters (Melles-Griot, US) and adjusted to 5.3 mW/cm^2^. The hUCM cells were irradiated once for five minutes (Green and RA + Green groups, separately), and one minute (Red and RA + Red groups, separately) at radiation energies of 1.59 J/cm² and 0.318 J/cm², respectively^1^ (Table 1). The spectrum of the LED device emission was tested by the spectrometer (Avantes, The Netherlands). All the exposures were carried out inside a CO_2_ incubator which was used for irradiation only. After the irradiation time was over, the plates were transferred into another CO_2_ incubator under the same conditions as the control samples (non-exposed cells). The experiments were replicated at least 3 (3–5) times under the same conditions.

**Table 1:** LED irradiation parameters.

Light sources	Wavelength	Bandwidth	Power density/irradiance	Exposure time	Energy density/dose
Red LED	630 nm	10 nm	5.3 mW/cm^2^	1 minute	0.318 J/cm²
Green LED	530 nm	20 nm	5.3 mW/cm^2^	5 minutes	1.59 J/cm²

### Induction of neural differentiation and Immunocytochemistry analysis

At the third passage, the cells were seeded at a density of 1 × 10^4^ cells/ml onto glass coverslips. After 24 h incubation at 37 °C, the hUCM cells in RA, RA + red and RA + green groups were treated with RA (10^-6^ M in 0.5% DMSO)^23^. Red and green groups did not receive RA but received irradiation as stated earlier. The induced cells in the different groups were cultivated for three weeks in DMEM/F12, supplemented with 10% FBS, as the basic medium. The medium was refreshed every 3 days.

At days 0, 7, 14 and 21 after the onset of treatments, the differentiation of hUCM cells was assessed by immunocytochemistry. The cells were washed with PBS and fixed in 4% paraformaldehyde for 40 min followed by two times washing in PBS. The cells were then permeabilized with PBS containing 0.1% Triton X-100, 1% bovine serum albumin and 10% normal goat serum for 40 min. Finally, the samples were washed with PBS and incubated overnight at 4 °C with anti-nestin (1/200,), anti-β-tubulin III (1/200,), anti-GFAP (1/200,) and anti-O4 (1/200,) antibodies. Afterward the cells were washed with PBS and incubated with anti-Mouse IgG-FITC for 90 min at room temperature. The cells were counterstained with Hoechst and the control slides were prepared by omitting the primary antibodies. Eventually, for each antibody and time point 200 cells were randomly counted and the proportion of antibody-stained cells was calculated according to the Hoechst-stained cells. Experiments were repeated three times. The cells were visualized by a fluorescent microscope (IX71, Olympus, Japan) equipped with a digital camera.

### RNA isolation and qRT-PCR

hUCM cells were cultured in 3 Cm petri dishes as described for immunocytochemistry. At days 0, 7, 14 and 21 of treatment, total RNA was extracted from hUCM cells using RNeasy kit (Qiagen, Crawley, UK) according to the manufacturer’s protocol. RNA integrity was evaluated and cDNA was synthesized using Omni script RT Kit (Qiagen). Quantitative polymerase chain reactions (qRT-PCR) were done in triplicate on each sample of cDNA. The PCR conditions were 10 min at 95 °C, followed by 35 cycles of 95 °C for 20 seconds for denaturation, and 60–65 °C for 50 seconds for annealing/extension. Negative controls (no cDNA) were used in all experiments. Analysis of relative gene expression data was performed using 2^−DD*C*T^ method described previously by Livak *et al*.^50^. The primers presented in the Table 2 were used in this study.

**Table 2:** Primers used for qRT-PCR.

Gene	Primer sequence	Annealing temp (°C)
Nestin	F: 5′-CTCAGGTCCTGGAAGGTCG-3′	64.9
	R: 5′-AAAGCTGAGGGAAGTCTTGGAG-3′	
β-tubulin III	F: 5′-CCCAGCGGCAACTACGTGGG-3′	61.6
	R: 5′-GTTGTTGCCGGCCCCACTCT-3′	
Olig2	F: 5′-AGACTCTCCTCAACTCGGCT-3′	60.6
	R: 5′-TGTTGTCGCTCCGACTTCTC-3′	
GAPDH	F: 5′-TGCACCACCAACTGCTTAGC-3′	60
	R: 5′-TGCACCACCAACTGCTTAGC-3′	

### Statistical analysis

The data are expressed as mean ± SD. Statistical analysis was performed by One-way ANOVA followed by Tukey post hoc test. P < 0.05 was considered statistically significant.
